# Cell attachment and proliferation of osteoblast-like MG63 cells on silk fibroin membrane for guided bone regeneration

**DOI:** 10.1186/s40902-016-0062-4

**Published:** 2016-03-30

**Authors:** Chae-Kyung Yoo, Jae-Yun Jeon, You-Jin Kim, Seong-Gon Kim, Kyung-Gyun Hwang

**Affiliations:** 1 Department of Translational Medicine, Graduate School of Biomedical Science and Engineering, Hanyang University, Seoul, South Korea; 2 Department of Dentistry/Division of Oral and Maxillofacial Surgery, College of Medicine, Hanyang University, 222 Wangsimni-ro, Seongdong-gu, Seoul, 133-791 South Korea; 3 Korean Minjok Leadership Academy, Hoengseong, South Korea; 4 Department of Oral and Maxillofacial Surgery, College of Dentistry, Gangneung-Wonju National University, Gangneung, South Korea

**Keywords:** Bone regeneration, Cell adhesion, Cell proliferation, Membrane, Osteoblasts, Silk fibroin

## Abstract

**Background:**

The aim of this study is to verify the feasibility of using silk fibroin (SF) as a potential membrane for guided bone regeneration (GBR).

**Methods:**

Various cellular responses (i.e., cell attachment, viability, and proliferation) of osteoblast-like MG63 cells cultured on an SF membrane were quantified. After culturing on an SF membrane for 1, 5, and 7 days, the attachment and surface morphology of MG63 cells were examined by optical and scanning electron microscopy (SEM), cell viability was determined using a 3-(4,5-dimethylthiazol-2-yl)-2,5-diphenyltetrazolium bromide (MTT) assay, and cell proliferation was quantified using 4′,6-diamidino-2-phenylindole (DAPI) fluorescence staining.

**Results:**

Optical microscopy revealed that MG63 cells cultured on the SF membrane proliferated over the 7-day observation period. The viability of cells cultured on SF membranes (SF group) and on control surfaces (control group) increased over time (*P* < 0.05); however, at respective time points, cell viability was not significantly different between the two groups (*P* > 0.05). In contrast, cell proliferation was significantly higher in the SF membrane group than in the control group at 7 days (*P* < 0.05).

**Conclusions:**

These results suggest that silk fibroin is a biocompatible material that could be used as a suitable alternative barrier membrane for GBR.

## Background

Several approaches for alveolar bone regeneration have been studied, including autogenous graft, allogenic graft, xenogenic graft, use of alloplastic materials, and distraction osteogenesis [1–3]. Guided bone regeneration (GBR) was introduced in the context of orthopedic research as early as 1959 [4]. The basic principles of GBR as a surgical procedure were developed by Melcher in 1976 and involve suppressing the growth of unwanted cellular tissues to create space for the growth of the desired tissue [5]. Features of an ideal membrane include easy handling, bio-absorbability, and biofunctionality [6, 7]. Membranes could additionally prevent epithelial cell movement to the bone defect site while permitting osteoblast migration to allow osteoblasts to carry out the regenerative process [8, 9]. Therefore, ideal membranes used in bone tissue regeneration should prevent local tissue cells of the sintered body and other epithelial cells from accessing the bone defect area [10, 11]. However, currently developed resorbable membranes are unable to achieve complete bone regeneration because of an induced inflammatory response caused by inadequate micro-environmental separation [12, 13]. Therefore, the development of a biocompatible membrane that resolves these current limitations is important and necessary.

Non-woven silk fibroin net is produced from the silk cocoon of the common silkworm moth, *Bombyx mori*. Silk fibroin (SF) has the ability to support the growth of different cell types, including endothelial, epithelial, fibroblast, glial, keratinocyte, and osteoblast cells [14]. It has also been applied in various biomedical applications: as a substrate material for tissue engineering scaffolds, in drug delivery, and even as artificial blood vessels because of its high tensile strength and low solubility in aqueous solutions [15, 16]. The physical and biological properties of silk fibroin make it an ideal candidate material for barrier membranes that resolve the limitations of previously developed membranes [17, 18].

Thus, the aim of this study is to evaluate various cellular responses of osteoblast-like MG63 cells to silk fibroin to verify the effectiveness and feasibility of silk fibroin as a potential membrane for GBR.

## Methods

### Cell culture on SF membranes

Osteoblast-like MG63 cells (ATCC, Manassas, VA, USA) were cultured 100-mm culture dishes ingrown in Dulbecco’s modified Eagle medium (DMEM, Gibco, USA) supplemented with 10 % fetal bovine serum (FBS, ATLAS, Dae Myung Science Co., Ltd., Korea) and 1 % penicillin-streptomycin (Pen-Strep, Gibco, USA). Cultures were incubated at 37 °C in a humidified atmosphere of 95 % air, and 5 % CO_2_, and the medium was changed every 2 days [19].

**Fig. 1:**
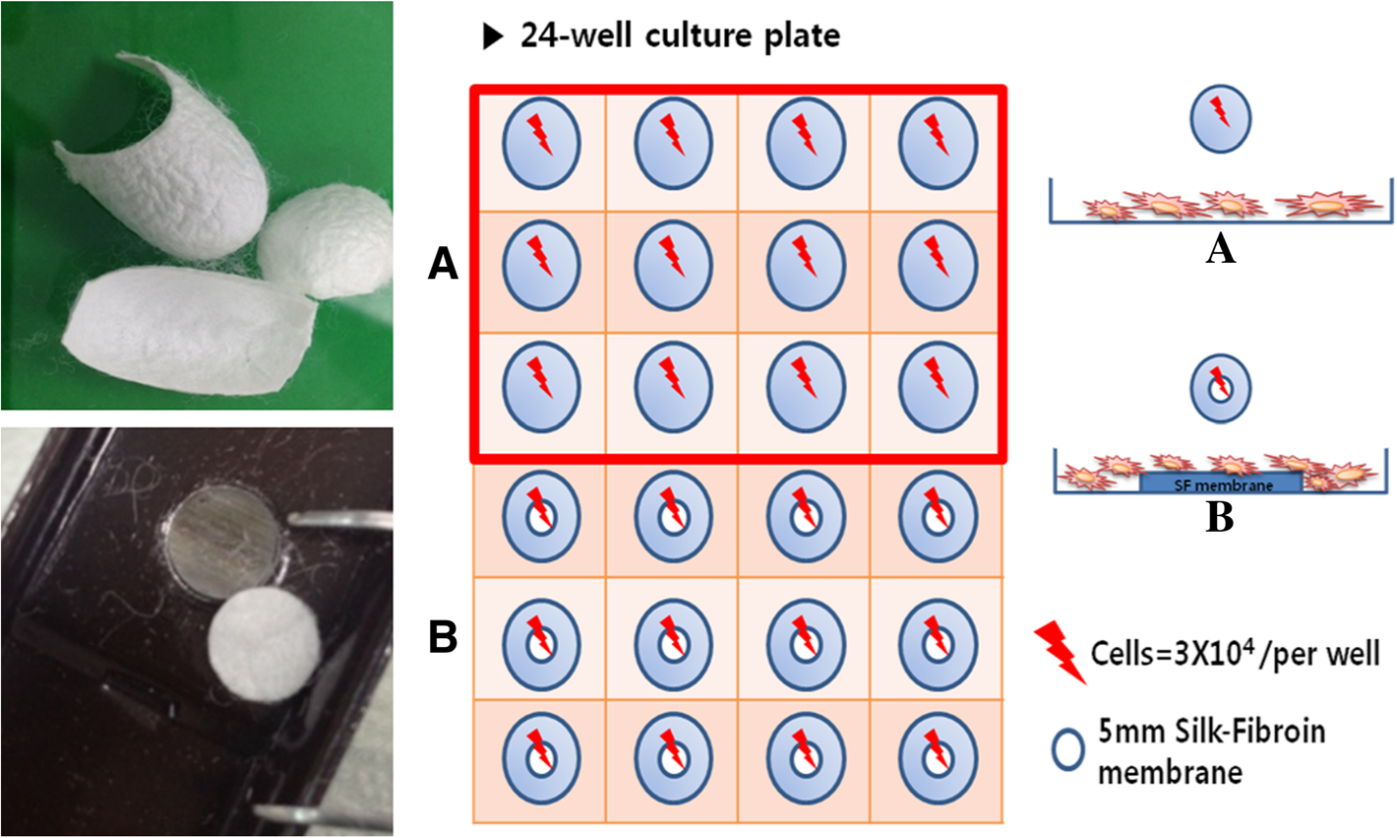
Silk membranes were prepared for culture with cells by cutting the film into discs 5 mm in diameter. Osteoblast-like MG63 cells (3 × 10^4^ cells/mL) was seeded onto a new 24-well plate with 1 mL fresh medium to each well. **a** Control group; **b** silk fibroin membrane group

SF membranes were prepared as previously described and stored in a sterile environment. Membranes were cut into 5-mm-diameter discs and sterilized by immersion in 70 % ethanol for 10 min followed by rinsing with phosphate-buffered saline (PBS). Sterile discs were placed in 24-well culture plates with spray glue, and culture medium was added to keep discs moist until cells were seeded. MG63 cells, cultured in 100-mm cell culture dishes, were trypsinized (0.25 % trypsin EDTA, Gibco, USA), and 3 × 10^4^ cells in 1 mL of fresh medium were seeded onto 24-well plates with or without prepared SF membranes (SF membrane group or control group, respectively; Fig. 1).

### Visualization of cell attachment

After culturing for 0, 1, 5, or 7 days, attached cells were imaged with optical microscopy (Leica DMI4000B, Leica, Germany) and scanning electron microscopy (SEM, Nova NanoSEM 450, FEI, USA) to compare the morphology of cells on the surface of SF membranes (SF membrane group) to those on culture plastic (control group). SEM imaging was performed after fixing cells with 4 % paraformaldehyde (Sigma-Aldrich, Inc., USA), dehydrating samples with graded ethanol (EMSURE® Ph Ethanol absolute for analysis) (70, 90, and 100 %), and further drying samples in an oven for 24 h. Fixed samples were sputter-coated with gold and imaged with the SEM [20].

### MTT assay for cell viability

The viability of cells cultured on SF membranes for various times up to 7 days was evaluated using thiazolyl blue tetrazolium bromide (MTT, Sigma-Aldrich, Inc., USA) following the manufacturer’s instructions. MTT reagent was added to each sample and incubated for 3 h to allow the formation of MTT formazan. The resulting formazan was educed with dimethyl sulfoxide (DMSO, Sigma-Aldrich, Inc., USA), and the absorbance of each solution was measured at a wavelength of 595 nm with a microplate reader (Bio-Rad, Japan) in triplicate. Cell viability was determined by comparing the absorbance of samples to a standard curve [21].

### DAPI staining for counting cell numbers

The number of cells was determined by 4′,6-diamidino-2-phenylindole (DAPI) (Vector Laboratories, Inc., Burlingame, CA, USA) fluorescence staining. Cells grown on SF membranes were washed with PBS (Gibco, USA) once before and twice after being fixed on the membrane with 4 % paraformaldehyde (Sigma-Aldrich, Inc., USA) for 15 min at room temperature. Membranes were then mounted on a slide, and the nuclei of cells were stained with DAPI and visualized and counted using an inverted fluorescence microscope (Leica DMI4000B, Leica, Germany) [22]. Visual field for cell counting was selected randomly.

### Statistical analysis

Each experiment was conducted at least thrice and replicated four times. All the data were expressed as the mean ± standard deviation for *n* = 4. The statistical difference was analyzed using Kruskal-Wallis one-way analysis of variance by ranks (IBM SPSS Statistics 21, IBM, USA), and a *P* value of <0.05 was considered significant.

## Results

### Cell attachment and proliferation on SF membranes

**Fig. 2:**
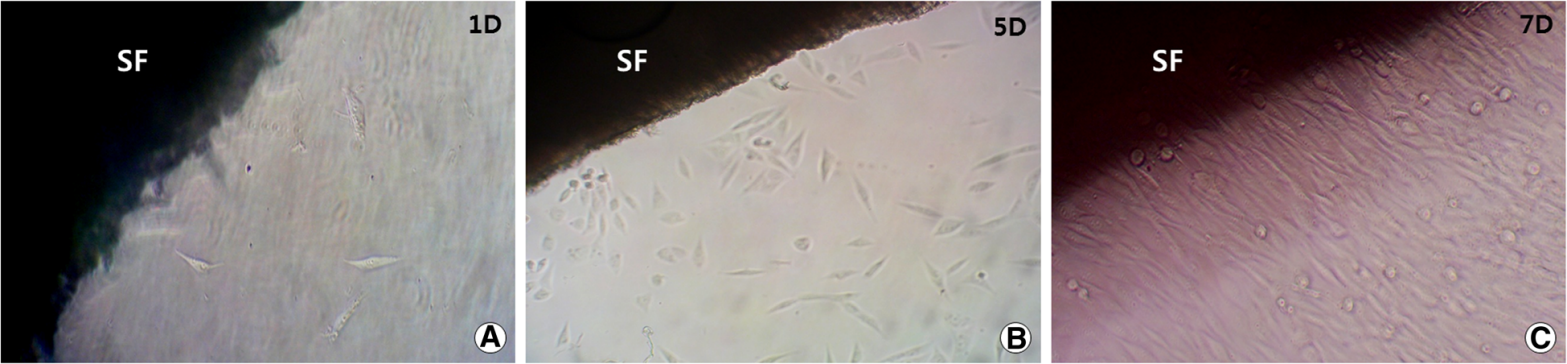
Cells multiplied and proliferated around the silk fibroin membrane from 1 to 7 days. **a** At day 1, the attachment of the cells to one or two peripheral membranes was confirmed. **b** At 5 days, the cells were attached around the membrane and had formed colonies. **c** At 7 days, the cells had proliferated and attached around the membrane to confluence (Optika ×10/0.25). *SF* silk fibroin membrane, *1D* 1 day after cell seeding, *5D* 5 days after cell seeding, *7D* 7 days after cell seeding

MG63 cells were seeded onto SF membranes (SF membrane group) or on culture plastic (control group) and assessed for differences in their proliferation over a range of 1 to 7 days (Fig. 2). MG63 cells were still in suspension 1 h after seeding; however, after 1 day, cells had adhered onto or grew adjacent to the membrane (Fig. 2a). After 5 days, cells were starting to form observable colonies (Fig. 2b), and after 7 days, colonies were larger and denser (Fig. 2c).

### Cell confluence on SF membranes

**Fig. 3:**
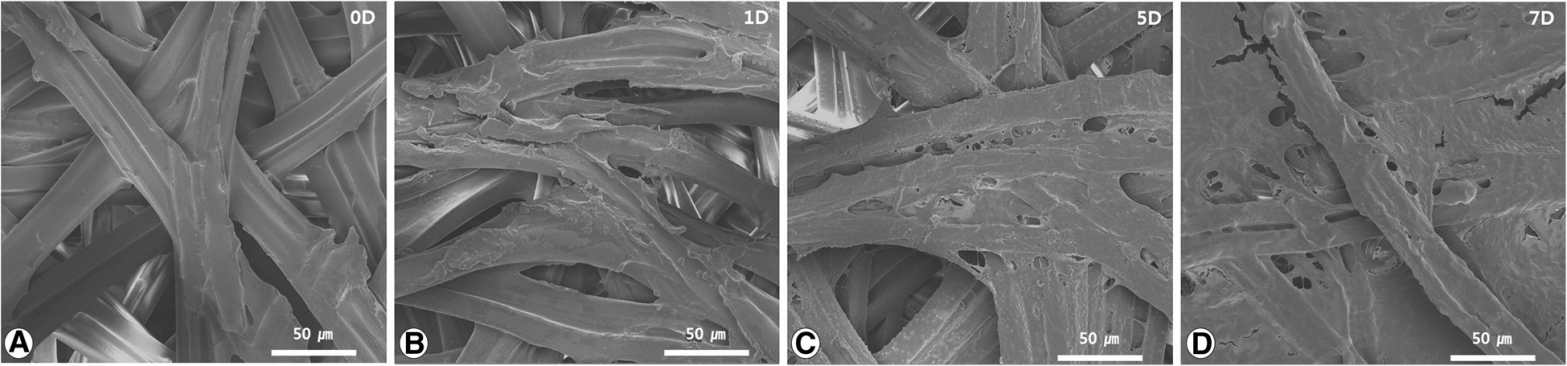
Scanning electron microscopy (SEM) images of cell attachment on the surface of the silk fibroin (SF) membrane. **a** SEM image shows the SF membrane surface for day 0, after seeding the initial cell number of 3 × 10^4^. **b** The cells gradually filled across the fiber texture covering approximately 10–20 % of the SF membrane surface. **c** At 5 days, the gap between the SF membrane fibers was filled and covered by the cell attachment. **d** At 7 days, the surface of the SF membrane was nearly filled and covered with cells, to about 90 % confluence, and the fiber texture was nearly totally obscured in the SEM image. *0D* cell seeding day, *1D* 1 day after cell seeding, *5D* 5 days after cell seeding, *7D* 7 days after cell seeding

After culturing MG63 cells on SF membranes for 0, 1, 5, or 7 days, membrane surfaces were imaged by SEM to determine cell confluence (Fig. 3). After 1 day, MG63 cells were 10–20 % confluent on the SF membrane surface compared with 0 day (Fig. 3a, b). After 5 days, MG63 cells were 50–60 % confluent (Fig. 3c), and by day 7, cells were 90 % confluent, nearly covering the entire surface of the membrane (Fig. 3d).

### Cell viability on SF membranes

**Fig. 4:**
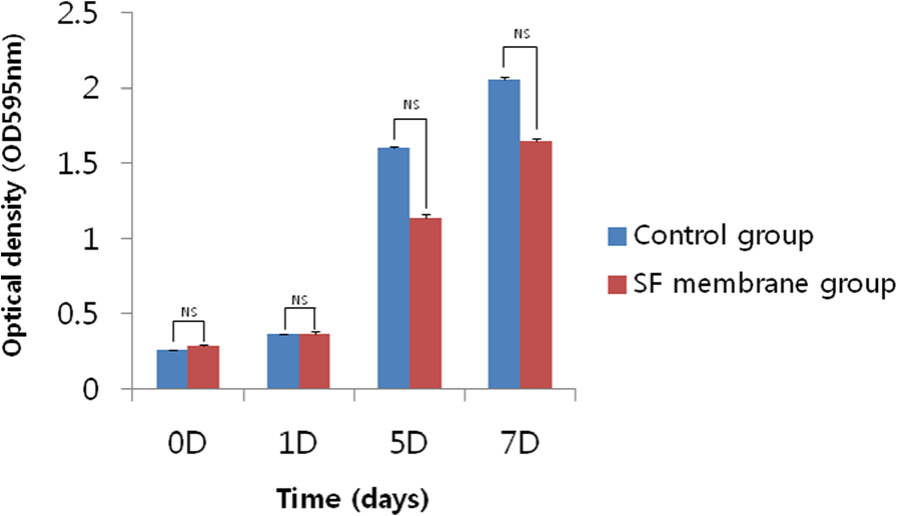
Evaluation of the cell viability by MTT assay using the optical density value. *OD* optical density, *SF* silk fibroin, *0D* cell seeding day, *1D* 1 day after cell seeding, *5D* 5 days after cell seeding, *7D* 7 days after cell seeding, *NS* not significant

Though the optical density values of formazan solutions from the SF membrane group were lower than those from the control group at respective time points (Fig. 4), these differences were not statistically significant (*P* > 0.05). This is consistent with our data that the proliferation of cells in the SF membrane group was similar to that of the control group at 7 days.

### Counting cell number on SF membranes

**Fig. 5:**
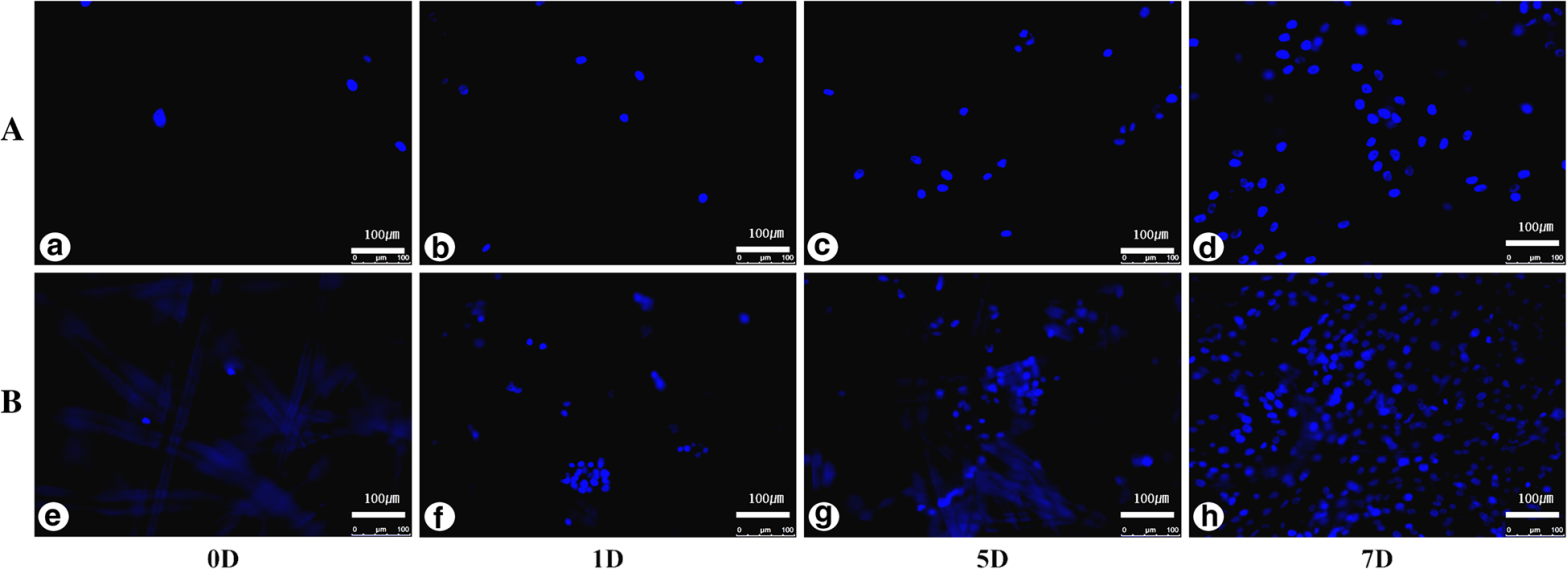
Cells stained by DAPI were counted over time. **A** control group (**a**-**d**), **B** silk fibroin membrane group (**e**-**h**). *0D* cell seeding day, *1D* 1 day after cell seeding, *5D* 5 days after cell seeding, *7D* 7 days after cell seeding

**Fig. 6:**
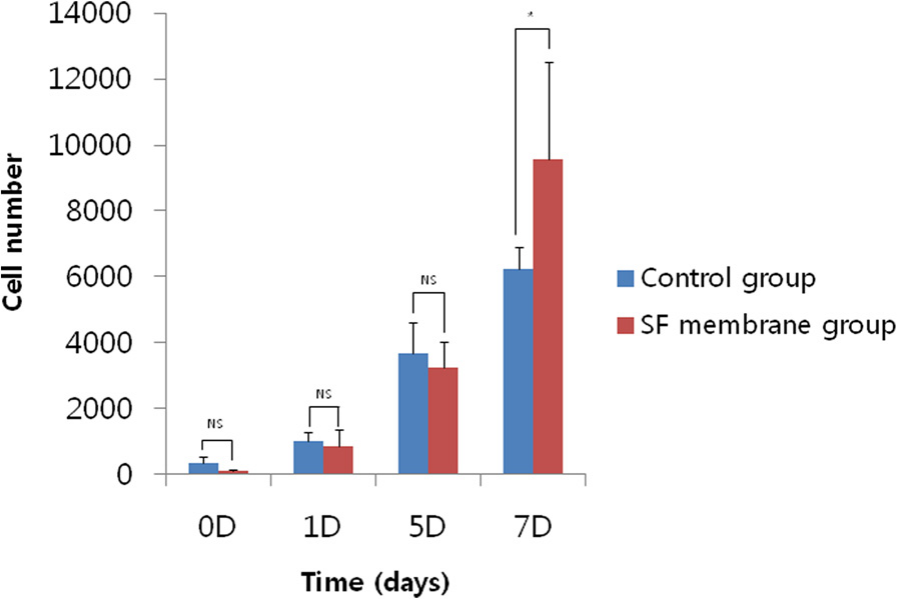
Comparing the number of cells between the both groups through DAPI staining. *SF* silk fibroin, *0D* cell seeding day, *1D* 1 day after cell seeding, *5D* 5 days after cell seeding, *7D* 7 days after cell seeding, *NS* not significant. The *asterisk* means significant at *P* < 0.05

To compare proliferation rates, the number of cells on SF membranes (SF membrane group) or on culture plastic (control group) was quantified over time by counting DAPI-stained nuclei (Fig. 5). We quantified the average number of cells in at least 10 photomicrographs, which were captured at various regions of the SF membrane including from the periphery to the center (Fig. 6). On day 0, 2.8-fold more cells adhered to the culture plastic (control group, 344 ± 180 cells) than to SF membranes (SF membrane group, 123 ± 33 cells). After 1 and 5 days, the fold difference in the number of cells between the control and SF membrane group was only 1.1, although the control group still had a greater number of cells. After 7 days, 1.6-fold more cells were counted in the SF membrane group (9821 ± 3351) than in the control group (6095 ± 848). Although the number of cells in both groups increased significantly over time (*P* < 0.05), differences in the number of cells between the control and SF membrane group were not significantly different (*P* > 0.05) at 0, 1, and 5-day points. The number of cells in the control and SF membrane group increased by 17.7 and 79.8 times, respectively, over 7 days (*P* < 0.05). Thus, cells proliferated 4.5-fold faster in the SF membrane group than in the control group, and the number of cells in the SF membrane group was significantly increased more than that in the control group at 7 days (*P* < 0.05).

## Discussion

In this study, we validated the biocompatibility of silk fibroin by showing that osteoblast-like MG63 cells can attach to, are viable on, and can proliferate on SF membranes. Silk fibroin is not only easily obtained from the common silkworm cocoon, but it is also recognized for its superior biocompatibility [23]. Osteogenic cells migrate to defective alveolar bone regions as part of the regenerative process, where a semi-permeable barrier membrane may assist in regulating the passage of specific biomolecules, such as growth factors that support angiogenesis, cytokines, and other nutrients [19, 24].

Attachment of MG63 cells onto the surface of SF membranes could not be verified by optical microscopy. However, the proliferation of cells adjacent to the membrane was confirmed. Additionally, adherent cells on SF membranes were observed by SEM analysis, which agrees with previous results. Kim et al. [18] showed the attachment of a confluent layer of cells on SF membranes with SEM, and Minoura et al. [25] confirmed the ability of silk fibroin nets to promote cellular attachment and growth with confocal microscopy and SEM.

Previous studies have also investigated the interaction of different cells with different membranes. Berahim et al. [26] observed the attachment and proliferation of fibroblasts on commercial collagen- and polyglycolic acid-based membranes with SEM over time. Carpio et al. [27] compared the attachment of cells on resorbable membranes (collagen membrane, glycolide fiber membrane) versus non-resorbable polytetrafluoroethylene (ePTFE) membranes with SEM. Approximately two times more cells attached onto the resorbable membrane than ePTFE. Additionally, a similar study by Wang et al. [28], in which they imaged osteoblast cells on six commercial membranes (BioMend, Resolut, GUIDOR, Epi-Guide, Gore-Tex, and Millipore filter) over time with SEM, had similar results as ours. Thus, silk fibroin has comparable cell attachment properties as other commercially manufactured membranes.

We investigated the proliferation and viability of MG63 cells on SF membranes using MTT assays. The growth rate of cells in the two groups was not significantly different (*P* > 0.05). Previous studies, which also used MTT assays, demonstrated that cell viability and proliferation were promoted by silk fibroin [18, 29]. Using MTT assays and measuring ALP activity, Cai et al. [30] showed that the proliferation of cells on silk fibroin was equal to or better than that of cells on other membranes tested. This is consistent with our results, which show that SF membranes do not negatively influence cell proliferation and viability.

The MTT assay could not be used to count the number of membrane-attached cells directly. SEM imaging was performed to demonstrate cell attachment, but it also could not be used to quantify the number of cells over time. Therefore, the number of cells was quantified by counting the number of DAPI-stained nuclei at various time points, which confirmed the proliferation of cells on SF membranes [31]. The number of cells in the SF membrane group counted after 7 days was greater with the DAPI-stained nuclei than by MTT assay. Whereas cells in the entire well (i.e., on and surrounding SF membranes) were included in MTT assays, only cells adhered onto SF membranes were included in the quantification of DAPI-stained nuclei.

SF membranes were prepared by first selecting silk fibroin following methods described in a previous study [29]. Although silk fibroin is water-absorbent, SF membranes will first float since considerable time is required for its saturation. Thus, we tested methods to adhere silk fibroin onto a 12-mm cover glass to resolve this issue. Double-sided tape has been used to affix SF membranes onto plates [21]; in that case, cell attachment is compromised due to the barrier of the rings and the thickness of the tape. Therefore, spray glue was used to affix SF membranes to the cover glass.

Silk fibroin has favorable biocompatibility, oxygen permeability, and cell attachment capabilities and can be provided cheaply through industrial manufacturing [17, 32, 33]. SF membranes are suitable for bone regeneration process because they can promote an osteoblast response with appropriate calcium deposition and nodule formation in vitro [34], and Sofia et al. [35] evaluated the use of silk fibroin for new bone regeneration in vivo in an animal study. On the basis of our results and previous studies, silk fibroin represents a comparable or better material for use as a barrier membrane for biomedical applications, including for guided bone regeneration.

## Conclusions

Within the limits of our results, it was confirmed that the silk fibroin had a good biocompatibility about cell attachment and proliferation. This study suggests that silk fibroin membrane would be useful as a barrier material for GBR.

## Abbreviations

GBR: guided bone regeneration
SEM: scanning electron microscope
SF: silk fibroin
